# A resazurin-based, nondestructive assay for monitoring cell proliferation during a scaffold-based 3D culture process

**DOI:** 10.1093/rb/rbaa002

**Published:** 2020-03-11

**Authors:** Xianghui Gong, Zhuqing Liang, Yongxing Yang, Haifeng Liu, Jing Ji, Yubo Fan

**Affiliations:** r1 Key Laboratory for Biomechanics and Mechanobiology of Ministry of Education, School of Biological Science and Medical Engineering, Beihang University, Beijing 100083, People’s Republic of China; r2 Beijing Advanced Innovation Centre for Biomedical Engineering, Beihang University, Beijing 102402, People’s Republic of China; r3 National Research Center for Rehabilitation Technical Aids, Beijing 100176, People’s Republic of China

**Keywords:** resazurin, nondestructive viable cell estimation, cytotoxicity, resazurin depletion, cell-based regenerative medicine

## Abstract

Development of viable cell estimation method without sacrificing proliferation and functions of cells cultured on regenerative biomaterials is essential for regenerative engineering. Cytotoxicity and depletion of resazurin are critical but often overlooked limitations that hindered applications of resazurin in viable cell estimation. The present work found that cytotoxicity and depletion of resazurin depended on cell concentration, resazurin concentration and resazurin incubation time. A simple strategy which only allowed cells to incubate with resazurin during each measurement was developed to eliminate negative effects of resazurin. This strategy was verified by monitoring proliferation of MC3T3-E1 preosteoblasts on poly(d,l-lactic acid) scaffold during a continuous 3D culture process for up to 21 days, comparing the accuracy with MTT assay which is a destructive assay with high sensitivity and accuracy and commonly used in regenerative engineering and comparing viability, proliferation and differentiation functions of MC3T3-E1, which were treated with/without this strategy for nondestructive evaluation. This method showed comparable linearity of standard curve and characteristics of growth curve to MTT assay. No major negative effects of this method on MC3T3-E1 viability and functions were found. Our work highlighted the importance of the concentration and incubation time of resazurin in designing application-specific nondestructive viability assay and would be helpful in improving the implanted medical devices as well as in regenerative engineering.

## Introduction

The scaffold-based 3D culture has shown great potential in regenerative medicine [1], pharmaceutical testing [2] and disease modeling [3, 4]. The information of cell growth kinetics and cell viability is necessary in exploring optimal culture conditions [5], quality control of a regenerative engineering process [6] and evaluating the performance of engineered tissues [7, 8]. Usually, viable cells on the surface or inside the 3D scaffold cannot be observed directly. The current assays for cell growth and cell viability commonly used in tissue engineering such as MTT assay [9], LDH detection [10], [3H]-thymidine incorporation [11] and CCK8 assay [12] are destructive or interfere with cell functions and make the follow-up culture process or clinical use impossible. Therefore, development of the nondestructive quantitative assay for viable cell estimation without sacrificing the precious patient-derived cells and disturbing cell functions is the urgent need for 3D culture process and regenerative engineering.

Resazurin is considered as a low cytotoxic dye and can be reduced by viable cells to resorufin [13]. The direct correlation between the resazurin reduction and the viable cell quantity was commonly used to assess the active growth of bacterial and cells [14]. Recently, resazurin-based viability assay was reported as a nondestructive assay to monitor cell proliferation on scaffolds or other biomaterials during a 3D culture process [15–17]. In these studies, resazurin was kept incubating with cells during the whole culture process. And the resazurin reduction was used to monitor the increase in viable cell number. However, several problems have yet to be resolved in these studies. (i) Although resazurin is generally accepted as a low toxic dye, several previous studies demonstrated the antiproliferative and cytotoxic effects of resazurin in leukemia cells and ovarian cancer cells [18, 19]. To our knowledge, limit work paid attention to changes of proliferation and functions of cells as well as the subsequent effects on tissue construction after resazurin exposure, all of which are very important factors to be considered in establishing a nondestructive method for viable cell estimation during a tissue construction process. (ii) High cell concentration and prolonged incubation time may lead to depletion of resazurin pool and loss of the accurate correlation between the resazurin reduction and the viable cell number, which is an important limitation often been overlooked. (iii) Resorufin can be furtherly reduced by living cells into hydroresorufin which is colorless and nonfluorescent [20]. This second redox step may lead to aberrant results that the system with high cell concentration produced a weak signal, while the system with low cell concentration yielded a high signal, particularly when the resazurin incubation time is long. To our knowledge, limited work evaluates effects of cell concentration and resazurin incubation time on cell viability, cell function and accuracy of a resazurin-based proliferation assay, all of which are critical to a 3D culture process.

In this study, we investigated changes of viability and functions of cells after resazurin exposure, which are very important factors to be considered in establishing a nondestructive method for viable cell estimation during a tissue construction process. Based on these investigations, we shared valuable insights not given elsewhere and provided a simple and efficient resazurin-based strategy for more accurately monitoring cell viability and cell growth without sacrificing cell viability and functions in a 3D culture process of up to 21 days.

## Materials and methods

### Scaffold fabrication

Scaffolds were fabricated from poly(d,l-lactic acid) (PLA, MW 400 kDa, Dikang Biomedical, Chengdu, China) using porogen leaching method. In brief, PLA/tetrahydrofuran solutions were mixed with 450–630-nm diameter potassium chloride (KCl) to obtain 93.74 ± 0.42% porosity (volume fraction) and dried in a 16-mm diameter glass mold at room temperature. Scaffolds (16 mm in diameter, 2-mm thick) were coated with 1 mg/ml gelatin solution for 10 h and crosslinked with 0.25% glutaraldehyde solution overnight. Then scaffolds were washed with ddH_2_O for 2 days (changed every 6 h) and dried in a lyophilizer. Scaffolds were disinfected by immersion in 75% ethanol for 4 h and washed with sterile PBS for six times. Then each scaffold (Supplementary Fig. S1) was cut into four equisized sectors. These sectors were used as scaffolds in the following experiments.

### Cell culture

The cell line of MC3T3-E1 preosteoblast was purchased from ATCC (Manassas, VA, USA). Stock cells were cultured in Alpha Minimum Essential Medium (α-MEM, Invitrogen, Carlsbad, CA, USA) supplemented with 10% fetal bovine serum (FBS, Gibco, USA) and 1% penicillin/streptomycin (Sangon, Shanghai, China). The rat aortic endothelial cell (RAEC) [21], rat bone marrow derived endothelial progenitor cells (EPCs) [22], rat bone marrow derived mesenchymal stem cell (MSC) [23] and human umbilical vein endothelial cells (HUVECs) [24] were isolated and cultured according to our previous studies. The RAECs were cultured in M199 medium (Gibco, Langley, OK, USA) containing 20% FBS, 1% penicillin/streptomycin and 50 μ/ml heparin (Ameresco, Solon, OH, USA). The EPCs were cultured with M199 medium containing 10% FBS, 50% endothelial cell medium (Sciencell, Carlsbad, CA, USA), 1% penicillin/streptomycin and 50 U/ml heparin. The MSCs were cultured in Dulbecco’s modified Eagle medium–low glucose (Gibco, Grand Island, NY, USA) supplemented with 10% FBS and 1% penicillin/streptomycin. The HUVECs were cultured in endothelial cell medium supplemented with 10% FBS. Cells were culture in cell incubator at 37°C with 5% CO_2_ and passaged routinely. Culture media were changed every 3 days. Cells were trypsinized upon subconfluence and counted by trypan blue staining. Aliquots of 40-μl cell suspensions (5 × 10^5^ cells/ml) were pipetted onto scaffolds. Each scaffold was seeded with 2 × 10^4^ cells. The cell-seeded scaffolds were cultured in 6-well plate for 24 h allowing cell adherence and then were incubated in 24-well plates with 1-ml culture media per well.

### MTT assay

Scaffolds were washed with phosphate buffered saline (PBS) two times and then incubated in 1 ml of fresh medium containing 20 µl MTT [3-(4,5-dimethyldiazol-2-yl)-2,5-diphenyl tetrazolium bromide; Sigma, St Louis, MO, USA, 5 mg/ml] solutions for 4 h. The medium was removed and 400 µl of 0.1 M HCl/10% sodium dodecyl sulfate (SDS) solution was added. The optical density (OD) of the sample was measured at 490 nm using a Varioskan Flash (Thermo Fisher Scientific, USA). Viable cells on the scaffolds were quantified using the standard curve, which was performed at each time point. All experiments were conducted in quadruplicate.

### Evaluating the cytotoxic effects of resazurin concentration and incubation time

MC3T3-E1 cells were seeded into 96-well plates at a density of 2 × 10^4^ cells/well and cultured for 24 h. Then cells were incubated in the media contained 2.0, 1.0, 0.4, 0.2 and 0.1 mM resazurin, respectively (100 μl/well). After 1, 2, 4, 8 and 24 h of incubation, the resazurin-contained media were removed. Cells were washed three times with PBS and cultured in fresh culture media for 48 h. Then cell viabilities were measured using MTT assay and normalized against with that of control cells, which were incubated in the fresh culture media. Each condition was tested in quadruplicate.

### Evaluating the effect of resazurin incubation time on viable cell estimation

MC3T3-E1 cells were seeded into 96-well plates at a density of 100, 50, 25, 12.5, 6.25, 3.12 and 1.56 × 10^4^ cells/well using double dilution method and cultured for 24 h. Then cells were incubated in the fresh media contained 0.2 mM resazurin (100 μl/well). The reduction of absorbance at 605 nm was measured by reading the absorbance of the sample on a Varioskan Flash at the time point of 0, 1, 2, 4, 8, 12, 24, 36 and 96 h. The color changes of the media were monitored by photograph at the same time intervals. Each condition was tested in quadruplicate. Wells contained no cells were used as control.

### Evaluating the residual resazurin on the cell-seeded scaffold after PBS washing

Four scaffolds were seeded with MC3T3-E1 cells at a density of 2 × 10^4^ cells/scaffold and cultured in 24-well plates. Culture media were changed every 3 days. After 7 days of culture, scaffolds were incubated in media containing 0.2 mM resazurin for 1 h. Then the resazurin was removed and the scaffolds were washed twice with PBS. At 0 and 1 h of the resazurin addition, and immediately after the PBS washing, color changes of the scaffolds were monitored by photograph. Then scaffolds were incubated in fresh media. At time intervals of 0, 1, 2 and 4 h, the release of the residual resazurin was determined by reading the absorbance at 605 nm using a Varioskan Flash. The non-cell-seeded scaffolds were used as control.

### Quantitative monitoring of cell viability and cell growth in the culture process

MC3T3-E1 cells were seeded onto the PLA scaffolds at a density of 2 × 10^4^ cells/scaffold and cultured in 24-well plates. During the culture, cell viability and cell growth were measured at 1, 4, 7, 10, 14, 18 21 days using resazurin assay. In brief, the scaffold was incubated with fresh media containing 0.2 mM resazurin in the cell incubator for 1 h. At 0 and 1 h of the resazurin addition, a 100-μl aliquot of sample solution was transferred to a new 96-well plate and assayed for the reduction of absorbance at 605 nm by reading the absorbance of the sample on a Varioskan Flash. Viable cells on the scaffolds were quantified using the standard curve, which was performed at each time point. After that, the scaffold was rinsed with sterile PBS two times to remove the resazurin and then cultured in fresh media in the cell incubator until the next measure time. The control group was not treated with resazurin and cultured by only changing media every 3 days. The viability and proliferation of the control group were measured using MTT assay in the same interval. Four cell-seeded scaffolds were used to conduct both the resazurin and MTT measurement at each time point.

RAECs were seeded onto the sulfated silk fibroin nanofibrous scaffold, which was prepared according to our previous work [21] at a density of 2 × 10^4^ cells/scaffold. MSCs and HUVECs were seeded in 24-well plates at a cell density 2 × 10^4^ cells/well, respectively. EPCs were seeded in 24-well plates at a cell density 1 × 10^4^ cells/well. All samples were cultured in 24-well plates. During the culture, cell viability and cell growth were measured at 1, 3, 5 and 7 days using resazurin assay as mentioned above. The control group was not treated with resazurin and cultured by only changing media every 3 days. The viability and proliferation of the control group were measured using MTT assay in the same interval.

### Live/dead cell viability assay

The viability of cells cultured on the scaffold was determined by live/dead staining at 7, 14 and 21 days of the incubation. The live/dead stain working solution was prepared by adding 10 µl of fluorescein diacetate stock (5 mg/ml, Sangon) and 10 µl of propidium iodide stock (2.5 mg/ml, Sangon) to 10 ml of Hank’s Balanced Salts Solution (HBSS) [21, 25]. The scaffold was rinsed with sterile HBSS two times and incubated in 500 µl of live/dead stain working solution at 37°C for 20 min. Then the scaffold was washed with HBSS twice and observed under fluorescence microscopy (Leica DM IRB, Germany). Viable cells fluoresce bright green, whereas dead cells fluoresce red owing to uptake of propidium iodide.

### RNA extraction and quantitative real-time PCR

Total RNA of MC3T3-E1 cell was isolated at 7, 14 and 21 days of 3D culture. At each time point, three scaffolds were pooled together as one sample. The scaffolds were gently rinsed with PBS to wash away residual media. The trizol reagent (Tiangen Biotech, Beijing, China) was directly added onto the scaffolds and gently agitated on a shaker for 20 min. The lysate together with the scaffolds was collected into Eppendorf tube followed by vortex and centrifugation. The clear cell lysate was transferred to a new RNAase-free Eppendorf tube. Then the total RNA isolation was performed according to the manufacturer’s protocol of RNAsimple total RNA kit (Tiangen Biotech). Gene expression of bone sialoprotein (BSP), collagen type I (COL I) and osteocalcin (OCN) was analyzed by quantitative real-time PCR. The first-strand cDNA was synthesized from 1 µg of total RNA using the ReverTra Ace (TOYOBO, Osaka, Japan). Real-time quantitative PCR was performed using RNA-direct SYBR Green Real-time PCR Master Mix (TOYOBO) in the Opticon PCR instrument (MJ Research, USA) (*n* = 3, total = nine scaffolds, three scaffolds were pooled). The cDNA amplified conditions was 60 s at 95°C and 5 s at 95°C, followed by 39 cycles of 15 s at 59°C and 30 s at 72°C, with data collection in the last 30 s. The relative mRNA expression was normalized against that of GAPDH using the 2^−ΔΔCq^ method [26]. Primer sequences were listed in Table 1.

**Table 1:** Primer sequences used for quantitative real-time PCR

Gene	Primer sequence (forward/reverse)
ALP	5′-GTTGCCAAGCTGGGAAGAACAC-3′
	5′-CCCACCCCGCTATTCCAAAC-3′
BSP	5′-TGTCTGCTGAAACCCGTTC-3′
	5′-GGGGTCTTTAAGTACCGGC-3′
COL	5′-TCCCTTGGACATTGGTGC-3′
	5′-AGTTTGGGTTGTTCGTCTGTT-3′
OCN	5′-AGGGAGGATCAAGTCCCG-3′
	5′-GAACAGACTCCGGCGCTA-3′
GADPH	5′-GTTGTCTCCTGCGACTTCA-3′
	5′-GGTGGTCCAGGGTTTCTTA-3′

### Alkaline phosphatase activity

At 4, 7, 10, 14, 18 and 21 days of incubation, cells cultured on the scaffold were rinsed two times with PBS followed by three cycles of freezing and thawing. The scaffolds were pipetted several times to enable cells lyse. Then alkaline phosphatase (ALP) activity was quantified using *p*-nitrophenyl phosphate (pNPP, Sigma) assay [16]. The pNPP solution was prepared according to Sun’s work. Aliquots of 50 µl cell lysate were added into 50 μl pNPP (1 mg/ml) solution and the mixture was incubated at 37°C for 15 min. The reaction was stopped by the addition of 25 μl of 3 M NaOH per 100 μl of reaction mixture. ALP activity was quantified by measuring the absorbance at 405 nm. The total protein content was determined using the BCA assay kit (Cwbio, Beijing, China) in aliquots of the same samples. ALP levels were normalized to the total protein content at the end of the experiment.

### Calcium content

At 4, 7, 10, 14, 18 and 21 days of incubation, calcium contents in the scaffold were examined by the orthocresolphthalein complexone (OCPC) method. The scaffolds were washed three times with PBS, immersed in 0.5 ml of 0.5 M acetic acid solution and incubated overnight. Then the scaffolds were pipetted several times to enable calcium release. Aliquots of 10 µl solution in the well were added into 300 µl of OCPC solution, and the mixture was incubated at room temperature for 10 min. Then the absorbance at 575 nm was measured using a Varioskan Flash. The OCPC solution was prepared according to van den Dolder’s work [27].

### NO measurement

At 3, 5 and 7 days of incubation, the culture medium of RAECs was harvested. The levels of NO were detected according to the manufacturer’s instructions of a commercial total NO assay kit (Beyotime Institute of Biotechnology, China), which is based on modified Griess reaction, and analyzed by reading the absorbance at 540 nm. The productivity of NO was obtained by normalizing the NO production of each sample with the incubation time of the corresponding culture media.

### Statistical analysis

Each experiment was repeated independently for at least 3 times. All data are expressed as mean ± SD (*n* = 3). Statistical analysis was performed using an unpaired two-tailed Student’s *t*-test. A value of *P* < 0.05 was considered statistically significant.

## Results

### Resazurin concentration and incubation time affect viability of MC3T3-E1 cell

**Figure 1:**
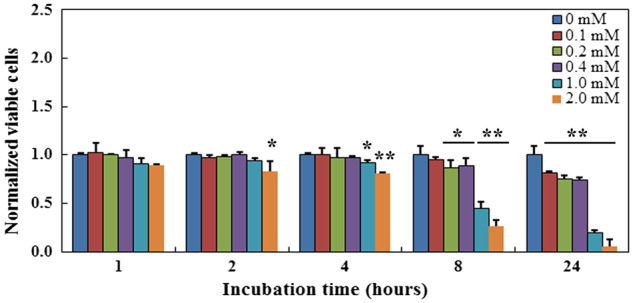
Cytotoxicity of resazurin concentration and incubation time to MC3T3-E1 cell. *Significant difference between the test group and the control group at *P* < 0.05. **Significant difference between the test group and the control group at *P* < 0.01

Effects of resazurin concentration and incubation time on viability of MC3T3-E1 cell were evaluated using the MTT assay (Fig. 1). No obvious decrease of viability was observed after 1 h of exposure to all resazurin-incubated groups compared with the control group (0 mM resazurin). Significant decrease of viability was observed with the increase of the incubation time (2.0 mM group at 2 h, 1.0 mM group at 4 h, 0.4 and 0.2 mM groups at 8 h and 0.1 mM at 24 h, *P* < 0.05), indicating that both resazurin concentration and incubation time affect viability of MC3T3-E1 cell. Moreover, after 24, 36 and 48 h of incubation with 0.1 mM resazurin, viability of cells decreased to 80.87 ± 2.49%, 71.79 ± 2.46% and 43.45 ± 4.54% of the control group, respectively (Supplementary Fig. S2), indicating cell damages resulting from the long-term resazurin incubation. As cell viability is very important to the performance of cell-based regenerative tissue implants, long-term incubation with resazurin, even under low concentration, should be avoided.

### Long-term resazurin incubation affects viable cell estimation through cytotoxic effects and resazurin depletion

**Figure 2:**
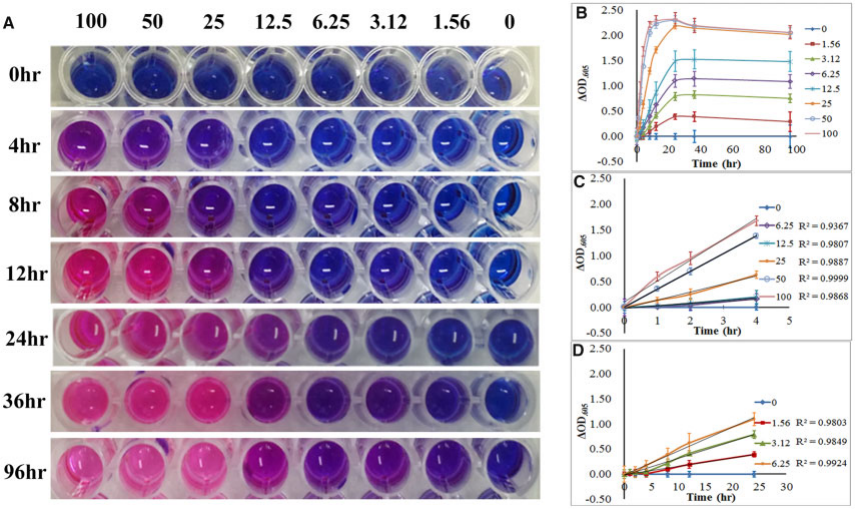
Effects of cell concentration and resazurin incubation time on viable cell estimation. (**A**) Graph of color changes of resazurin at different time point. (**B**–**D)** Absorbance reduction of resazurin as a function of viable cell concentration of MC3T3-E1 and incubation time. (B) The cell concentration ranged from 0 to 100 × 10^4^ cells/well, (C) the cell concentration ranged from 6.25 to 100 × 10^4^ cells/well and (D) the cell concentration ranged from 1.56 to 6.25 × 10^4^ cells/well

No significant decreases in cell viability were observed when cells were incubated with 0.1, 0.2 or 0.4 mM resazurin within 4 h (Fig. 1). We also found that MC3T3-E1 exhibited a better linearity to resazurin reduction over a wide cell concentration range and long reaction time when they were incubated with 0.2 mM resazurin than that incubated with 0.1 mM resazurin (Fig. 2 and Supplementary Fig. S3). Similar results were obtained from the RAEC, EPC, MSC and HUVEC (data not shown). Therefore, we used 0.2 mM resazurin in viable cell estimation.

We evaluated the effect of cell concentration and incubation period on viable cell estimation (Fig. 2). In the early stage of the incubation, the change in absorbance at 605 nm appeared to increase linearly with the increase in the incubation time. The linearities were good within 4 h in high cell density (6.25–100 × 10^4^ cells/well, Fig. 2C) and within 24 h in low cell density (1.88–3.12 × 10^4^ cells/well, Fig. 2D). Then, the change rate of the absorbance at 605 nm slowed until no obvious change was observed after 24 h of incubation in all cell concentration groups (Fig. 2B). Notably, for the cell density ranged from 25 to 100 × 10^4^ cells/well, the pink color of the media did not change obviously after 12 h of incubation, indicating depletion of the resazurin (Fig. 2A). For the cell density ranged from 1.88 to 3.12 × 10^4^ cells/well, the purple color of the media did not change obviously after 24 h of incubation, indicating enough resazurin remained in the well (Fig. 2A). Combined with results mentioned above (Fig. 1), the reduced rate of resazurin reduction in the low cell concentration group was due to the cytotoxicity of long-term resazurin incubation. Thus, long-term exposure to resazurin results in cytotoxicity to MC3T3-E1 cell and depletion of resazurin, both of which may contribute to failure of continuous viable cell estimation.

### Addition and removal of resazurin did not affect cell viability

**Figure 3:**
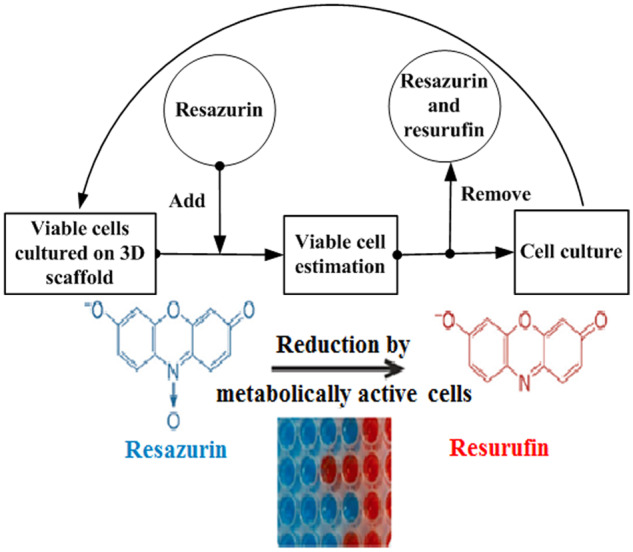
The schematic diagram of our strategy for continuous measurement of viable cells informed by the present study

We developed a new resazurin-based viable cell quantification strategy to avoid negative effects caused by high concentration and long incubation time of resazurin (Fig. 3). Resazurin was added into the culture system just before the measurement and incubated with viable cells for 1 h. Then the decrease of absorbance at 605 nm was measured. The resazurin and its product, resorufin, were removed by PBS washing immediately after the measurement. The cells were cultured in fresh medium until the next measurement.

**Figure 4:**
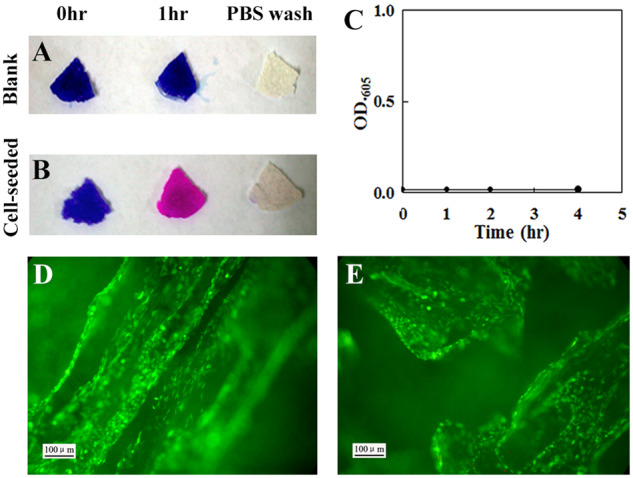
Addition and removal of resazurin did not affect the viability of MC3T3-E1 cultured on 3D PLA scaffolds. Color changes in the blank scaffold (**A**) and cell-seeded scaffold that were incubated for 7 days (**B**) after 0 and 1 h of the resazurin addition and immediately after the PBS washing (from left to right). (**C**) Evaluation of residual resazurin on the blank and cell-seeded scaffold after PBS washing by reading the absorbance at 605 nm. Each point was the mean of absorbance readings of four scaffolds. (**D** and **E**) Images of effects of the addition and removal of resazurin on cell viability evaluated by live (green)/dead (red) staining. (D) The control group and (E) the resazurin-incubated group. Scale bar: 100 μm

Whether the resazurin and its product can be removed from the porous scaffold was evaluated. At the 0 h of resazurin addition, both the blank scaffold and the cell-seeded scaffold were blue. After 1 h of resazurin addition, the blank scaffold was still blue, while the cell-seeded scaffold was pink, which indicated that resazurin was reduced to resorufin by viable cells. After PBS washing, both the blank scaffold and the cell-seeded scaffold became white, indicated that resazurin and resorufin were removed (Fig. 4A and B). After PBS washing for 0, 1, 2 and 4 h, the absorbance at 605 nm of the media incubated with the blank or cell-seeded scaffold was nearly 0 and almost identical (Fig. 4C), suggesting that no resazurin released from the cell-seeded scaffold after PBS washing. Moreover, viable cells on the resazurin-treated scaffold and untreated scaffold were visually determined by live/dead staining. No major difference in terms of cell viability was observed between these two groups (Fig. 4D and E). These results demonstrated that resazurin and resorufin could be easily removed from the scaffold by PBS washing and this process did not affect the viability of MC3T3-E1 cells.

### Effects of repeated viable cell measurement on cell viability and cell growth

**Figure 5:**
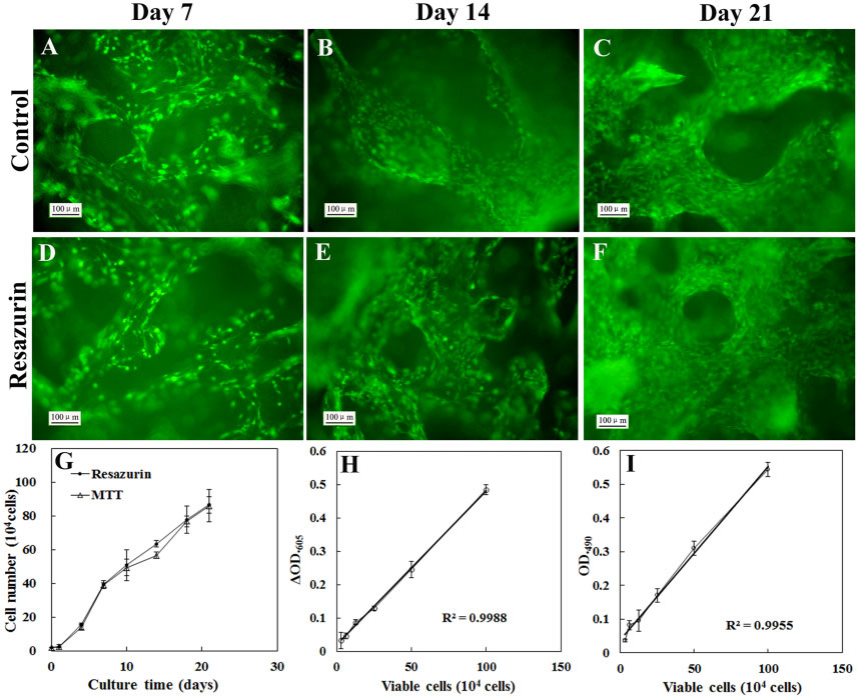
Effects of repeated viable cell estimation on viability and growth of MC3T3-E1 cultured on 3D PLA scaffolds. (**A**–**F**) The representative images of viable cells which were cultured on 3D PLA scaffold and determined by live (green)/dead (red) staining. (A–C) Viable cells of the control group. (D–F) Viable cells of the test group which was monitored by resazurin assay repeatedly. Scale bar: 100 μm. (**G**) The growth curves of MC3T3-E1 cells which were cultured on 3D PLA scaffold. The control group and the test group were monitored by MTT assay and resazurin assay, respectively. Each point was the mean of absorbance readings of four scaffolds. The representative standard curves of resazurin assay (**H**) and MTT assay (**I**)

Next we evaluated effects of repeated viable cell estimation of our strategy, which needs repeated addition and removal of resazurin during the 3D culture process, on cell viability and cell growth. Viable cells on the scaffold were determined by resazurin assay at 1, 3, 7, 10, 14, 18 and 21 days of incubation. The resazurin was added into the culture system just before the measurement and removed immediately after the measurement finished. Between the measurements, the cell-seeded scaffolds were cultured in fresh media. Viable cells on the control scaffolds were measured by MTT assay. The resazurin and MTT method exhibited comparable linearity of standard curve (Fig. 5H and I). The growth curve obtained by resazurin assay showed similar characteristics to that obtained by MTT assay. After 24 h of lag phase, cells grew exponentially from Day 2 to Day 7, and then viable cells increased slowly. At 21 days of incubation, viable cells on the scaffold achieved 85 × 10^4^ cells (Fig. 5G). At 7, 14 and 21 days of incubation, cell viability and proliferation were visually determined by live/dead staining (Fig. 5A–F). The viable cell density appeared to increase with the prolongation of the culture time. Viable cell densities at 21 days of both groups were much higher than that at 7 and 14 days. Nearly no dead cells were observed. No major difference in terms of cell viability and cell proliferation was observed between the resazurin-incubated group and the control group. These results indicated that resazurin assay was comparable to MTT assay in sensitivity and reliability, and repeated addition and removal of resazurin did not affect cell viability and cell growth.

### Effects of repeated viable cell measurement on gene expression

**Figure 6:**
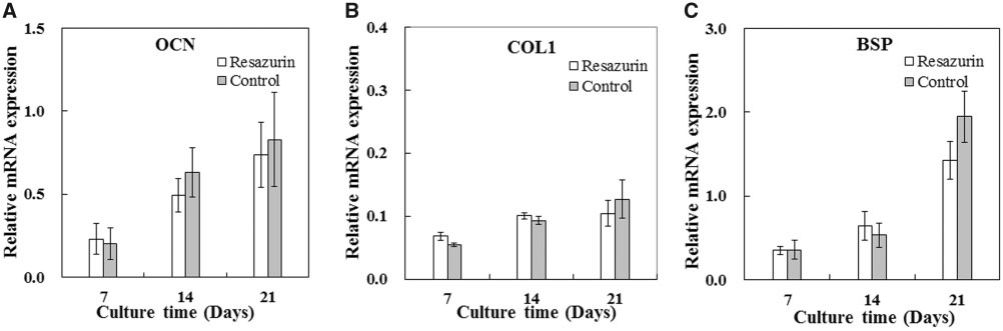
Effects of repeated viable cell measurement on gene expression of MC3T3-E1 cells cultured on 3D PLA scaffolds. Gene expression of BSP (**A**), COL I (**B**) and OCN (**C**) were analyzed by quantitative real-time PCR. Cells grown on scaffolds of the test group and the control group for 7, 14 and 21 days were used as samples. The *y*-axis represents fold changes of gene expression normalized by GAPDH. Each data was the mean of three samples, and three scaffolds were pooled as a sample (*n* = 3, total = nine scaffolds for one data)

To investigate effects of repeated viable cell estimation on function of MC3T3-E1 cells, expression of three marker genes, BSP, COL I and OCN, at 7, 14 and 21 days of incubation was analyzed by quantitative real-time PCR. All gene expression increased with culture time (Fig. 6). Although the gene expression of resazurin-incubated group at Day 21 was slightly less than that of the control group, the result of statistical analysis showed no significant difference in expression of BSP (*P* = 0.479 at Day 7, *P* = 0.220 at Day 14 and *P* = 0.094 at Day 21), COL I (*P* = 0.060 at Day 7, *P* = 0.124 at Day 14 and *P* = 0.219 at Day 21) and OCN (*P* = 0.393 at Day 7, *P* = 0.198 at Day 14 and *P* = 0.373 at Day 21) between the resazurin-incubated group and the control group (Fig. 6A–C), suggesting that repeatedly addition and removal of resazurin had little major effects on expression of these genes.

### Effects of repeated viable cell measurement on calcium deposition

**Figure 7:**
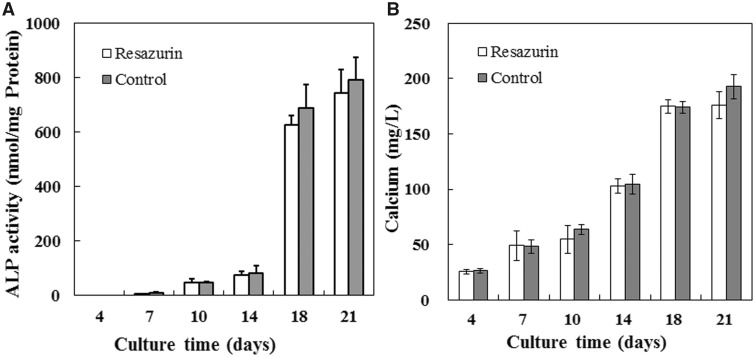
Effects of repeated viable cell measurement on ALP activity and calcium contents of MC3T3-E1 cells cultured on 3D PLA scaffolds. (**A**) The ALP activity of the test group and the control group cultured for 4, 7, 10, 14, 18 and 21 days. (**B**) Calcium contents of the test group and the control group cultured for 4, 7, 10, 14, 18 and 21 days. Each data was the mean of four scaffolds

We further investigate effects of repeated viable cell estimation on ALP activity and calcium deposition, which are important functions of osteoblasts. Both the ALP activity and the calcium deposits increase with the prolongation of the culture time (Fig. 7). No significant difference in ALP activity (*P* = 0.069 at Day 4, *P* = 0.207 at Day 7, *P* = 0.923 at Day 10, *P* = 0.989 at Day 14, *P* = 0.841 at Day 18 and *P* = 0.393 at Day 21) and calcium deposit (*P* = 0.719 at Day 4, *P* = 0.442 at Day 7, *P* = 0.527 at Day 10, *P* = 0.980 at Day 14, *P* = 0.978 at Day 18 and *P* = 0.741 at Day 21) according to the result of statistical analysis, although both the ALP activity and the calcium deposit of the test group at Day 21 were slightly less than that of the control group, which indicated that repeatedly addition and removal of resazurin had little major effects on the function of calcium deposition.

### Applications of the newly developed method to other cell types

**Figure 8:**
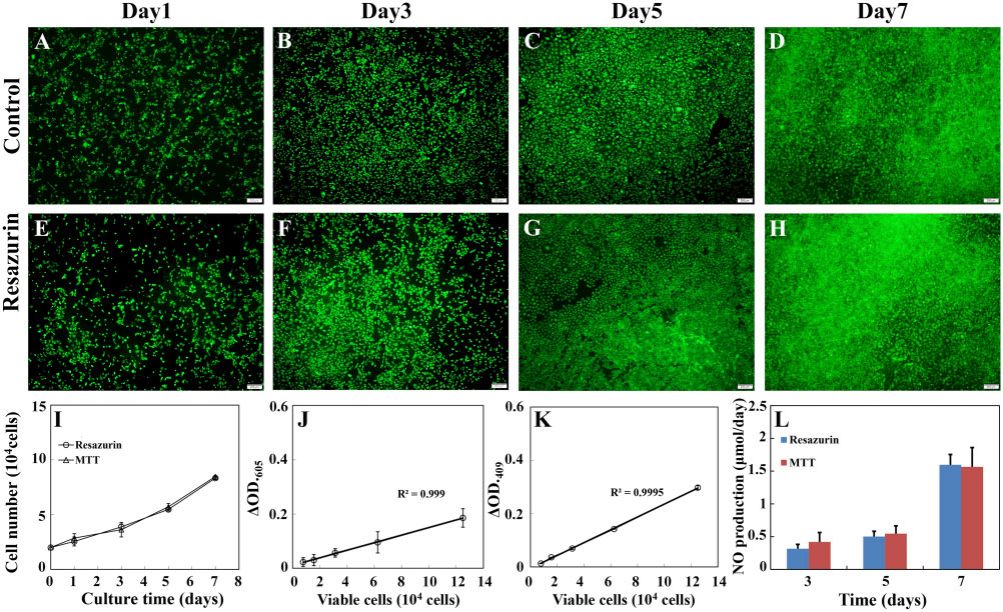
Effects of repeated viable cell estimation on viability, growth and hemostatic function of RAECs cultured on the sulfated silk fibroin nanofibrous scaffold. (**A–H**) The representative images of viable cells that were cultured on the sulfated silk fibroin nanofibrous scaffold and determined by live (green)/dead (red) staining. (A–D) Viable cells of the control group. (E–H) Viable cells of the test group, which was monitored by resazurin assay repeatedly. Scale bar: 200 μm. (**I**) The growth curves of RAECs, which were cultured on the sulfated silk fibroin nanofibrous scaffold. The control group and the test group were monitored by MTT assay and resazurin assay, respectively. The representative standard curves of resazurin assay (**J**) and MTT assay (**K**). (**L**) The productivity of NO of the test group and the control group cultured for 3, 5 and 7 days. Each data was the mean of four scaffolds

To demonstrate versatility of the newly developed method, the resazurin assay mentioned above was adapted to monitor RAECs growth during a 7-day long continuous culture without harming cells. RAECs were cultured on the sulfated silk fibroin nanofibrous scaffold. The cell growth was monitored at 1, 3, 5 and 7 days of incubation using resazurin assay as mentioned above. Similar to the results obtained from MC3T3-E1, no major differences in terms of viability (Fig. 8A–H), proliferation (Fig. 8I–K) and NO productivity (Fig. 8L), which is an important index of hemostatic function of RAECs, were observed between the resazurin-incubated group and the control group.

**Figure 9:**
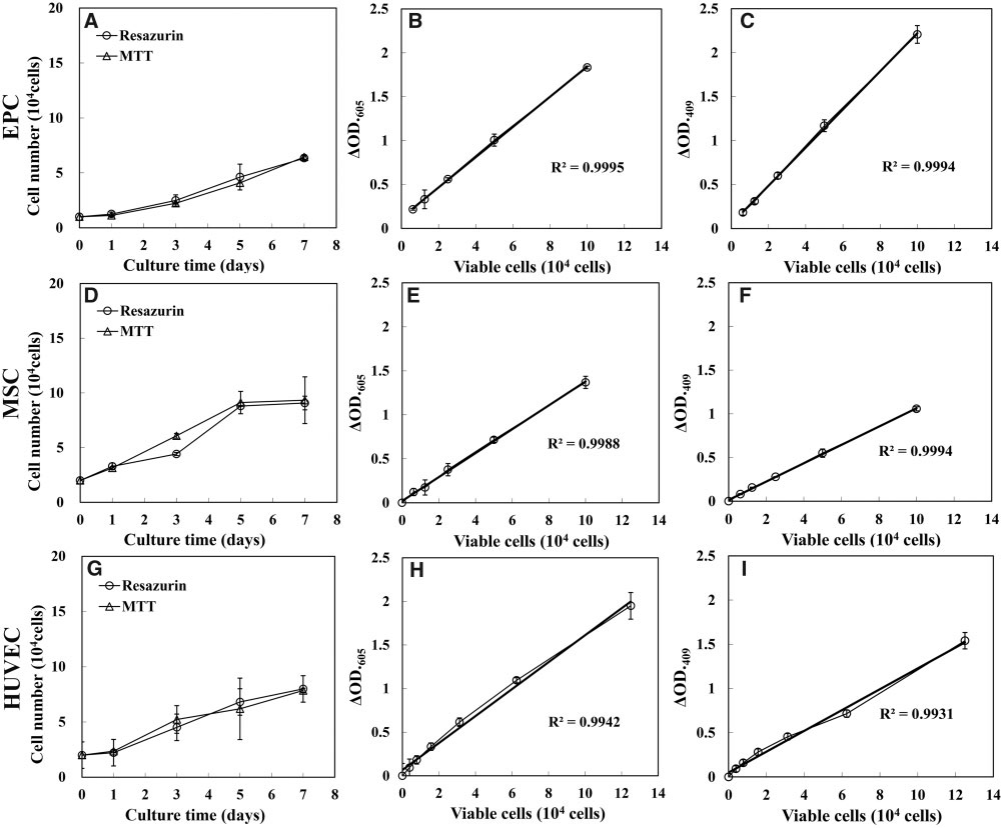
Effects of repeated viable cell estimation on growth of EPC, MSC and HUVEC cultured on 24-well plate. Growth curves of EPC (**A**), MSC (**D**) and HUVEC (**G**), which were cultured in 24-well plate. The control group and the test group were monitored by MTT assay and resazurin assay, respectively. Each point was the mean of absorbance readings of four scaffolds. The representative standard curves of resazurin assay of EPC (**B**), MSC (**E**) and HUVEC (**H**). The representative standard curves of MTT assay of EPC (**C**), MSC (**F**) and HUVEC (**I**)

Other three kinds of cells, EPC, MSC and HUVEC, with different intrinsic metabolic activity profiles and usages were adapted to evaluate the versatility of the newly developed method as a nondestructive assay for a continuous culture process furtherly. The resazurin and MTT method exhibited comparable linearity of standard curve. The growth curve of these cells obtained by resazurin assay showed similar characteristics to that obtained by MTT assay (Fig. 9).

## Discussion

A quantitative assessment of cell viability and cell growth without ending the cell culture process is particularly important in optimizing 3D culture conditions and quality control of cell-based regenerative medicine [17, 20]. It offered the advantages of keeping the cells in culture to observe changes over time and allowing further investigations or applications to be carried out on the same sample. Resazurin is considered as a good reporter dye for viable cell measurement [13, 14]. However, cytotoxicity, resazurin depletion and second redox step caused by high resazurin concentration and long incubation time are critical but often overlooked limitations that hindered its application in nondestructive cell viability and cell proliferation monitoring. The present study investigated effects of concentration and incubation time of resazurin on viability and functions of MC3T3-E1 with different cell densities and provided a resazurin-based assay for monitoring cell growth quantitatively without sacrificing cells or disturbing cell function during a 3D culture process.

MC3T3-E1 preosteoblast was used for investigation in this study. Osteoblastic differentiation of MC3T3-E1 usually requires weeks *in vitro*. Viable cell estimation with our strategy can be conducted many times during the long culture time. For example, viable cells were measured seven times during the continuous culture up to 21 days in this study. Therefore, it is a good model to verify the effectiveness and safety of our strategy by comparing the accuracy with MTT assay and evaluating effects of repeated adding and removal of resazurin on cell proliferation and function.

The present study demonstrated that long-term exposure to resazurin results in cytotoxicity to MC3T3-E1 cell and depletion of resazurin. Our data were supported by Erikstein’s report that the proliferation of HL-60 cells was significantly reduced after 6 h even in the lowest resazurin concentration (22 mM). They also found the attenuating effect of resazurin on proliferation of several primary AML blasts isolated from patients [18]. It is generally accepted that resazurin is a low toxic dye [13, 17, 20]. However, it is important to note that resazurin is low toxic, not nontoxic to cells. The present study found that the cytotoxicity of resazurin depends on its concentration and incubation time with cells. The cytotoxicity of resazurin might not have much influence on measuring the number of viable cells during long-term incubation when a standard curve was conducted under the same condition to identify the relationship between the change of absorbance (or fluorescence intensity) and the cell number and to eliminate systematic errors caused by cytotoxicity and second redox of resazurin. However, the cytotoxicity of resazurin does harm cell viability and functions, both of which are critical to the performance of the constructed tissue implants, and should be avoided. Limit work investigated the harm of resazurin cytotoxicity to proliferation and functions of cells as well as the subsequent effects on tissue construction when resazurin was used in viable cell estimation in tissue construction. Moreover, we demonstrated in this study that both of cytotoxicity and depletion of resazurin during long-term resazurin incubation reduced the rate of resazurin reduction or resorufin production. Previous studies have shown the fact that resazurin reduction reached a plateau and did not related to viable concentration when high cell concentration reached or incubation times prolonged (9–96 h of incubation) during continuous viable cell estimation [15, 28]. It is worth noting, however, that the resazurin-based assay previously used by others to relate colorimetric or fluorescence changes to viable cell number presents implicitly assumes that the rate of resazurin reduction or resorufin production does not change over the incubation period [13]. This can potentially result in a significant underestimation of cell number. Therefore, our data highlighted the importance of avoiding cytotoxicity and depletion of resazurin when it was used as a nondestructive viability reporter for 3D culture and regenerative medicines.

Previous studies reported that resazurin entered the cytosol of viable cells and was reduced by mitochondrial enzyme to resorufin. Resorufin could be transferred out of cells and easily measured by colorimetric or fluorometric reading [13, 29, 30]. Based on these studies, we developed a simple strategy to minimize the negative effect of cytotoxicity and depletion of resazurin caused by long resazurin incubation period and high cell concentration in this study. Resazurin was added into the culture system for viability assay and removed immediately after each measurement. The reaction time was optimized to make the resazurin reduction within the linear range. To our knowledge, this strategy has not been previously documented.

Our data showed that resazurin and its reduced product, resorufin, were removed easily by PBS washing. The addition and removal of resazurin, together with short-term incubation with cells (e.g. 1 h in the present study), did not affect cell viability significantly. These results enabled elimination of negative effects of the cytotoxicity, resazurin pool depletion and second reduction of resazurin caused by long-term incubation, which are important but often overlooked limitations that hindered the use of resazurin in invasive and quantitative viability monitoring process during 3D culture. Previous studies have used high concentration or increased working volume of resazurin, in order to avoid resazurin depletion and maintain the correlation between viable cell numbers and the rate of resazurin reduction or resorufin generation in the entire duration of the assay [20]. However, high concentration of resazurin suggests potential cytotoxicity and sacrifice of cell function, particularly during the long-term incubation. and a proper ratio of cell to resazurin working volume for maintaining good linearity between the viable cell and resazurin reduction required application-specific optimization, which consumes a huge amount of scaffolds and precious cells.

The present study evaluated effects of repeated addition and removal of resazurin on proliferation and differentiation of MC 3T3E1 cell, which are critical to bone regeneration. Our data showed comparable characteristics of standard curve and growth curve, and similar cell viability to those of MTT assay, which was the golden standard used in cell proliferation assay. These results demonstrated that repeated addition and removal of resazurin did not affect cell proliferation and cell viability. ALP, BSP, OCN and COL1 are molecules associated with mineralization and markers of osteogenic differentiation [31, 32]. Cells under repeated addition and removal of resazurin showed a decrease in the relative mRNA expression levels of ALP, BSP, OCN and COL1 than the control group, although we did not found significant differences between them. Resazurin has been reported to promote cellular ROS production, which results in suppression of differentiation markers such as ALP activity, COLI gene expression and the mineralization of osteoblastic cells [18, 33–35]. ROS production was triggered immediately after resazurin addition to the cell cultures, while the mitochondrial impairment and cell death were observed after long period of incubation [18]. These studies offered possible reasons for our data that short-term resazurin incubation had negative effects on differential function of MC 3T3E1 but did not affect cell proliferation. In addition, investigators of previous study found that cellular sensitivity to resazurin varied according to cell type and incubation conditions such as resazurin concentration and incubation time [18, 36, 37]. Therefore, the present study underscored the importance of carefully considering the concentration and incubation time of resazurin when it is employed in measuring living cells and provided a valuable strategy for resazurin-based nondestructive viability assay in 3D culture process.

## Conclusion

Our present data demonstrated that high concentration and long incubation time of resazurin lead to cytotoxic effects to MC3T3-E1 and resazurin depletion and strongly affect the effectiveness and accuracy of the resazurin-based nondestructive viable cell estimation. In order to overcome pitfalls mentioned above, we provided a simple and efficient strategy in which resazurin was incubated with viable cells only during each measurement period. This strategy showed comparable sensitivity and reliability to MTT assay and had no major negative effects on viability, proliferation and differentiation of MC3T3-E1 cell. Four types of cells, RAEC, EPC, MSC and HUVEC, were used to verify the versatility of this strategy as a nondestructive assay for a continuous culture process, and results similar to MC3T3-E1 were obtained. Our work would benefit the research, manufacture and evaluation of cell-based regenerative medicine by allowing monitoring viability and proliferation of cells without sacrificing tissue construction.

## Supplementary Material

rbaa002_Supplementary_Materials
