# Supplementary material for: A resazurin-based, nondestructive assay for monitoring cell proliferation during a scaffold-based 3D culture process
Source: Regen Biomater. 2020 Mar 11;7(3):271–81. doi: 10.1093/rb/rbaa002 (PMC7266666; doi:10.1093/rb/rbaa002)
Supplement: rbaa002_Supplementary_Materials [file rbaa002_supplementary_materials.doc]

**Supporting Information for**

A Resazurin-based, Nondestructive Assay for Monitoring Cell Proliferation During a Scaffold-based 3D Culture Process

Xianghui Gong^ab*^, Zhuqing Liang^ab^, Yongxing Yang^a^, Haifeng Liu^a^, Jing Ji^a^, Yubo Fan^a,b,c*^

^a^Key Laboratory for Biomechanics and Mechanobiology of Ministry of Education, School of Biological Science and Medical Engineering, Beihang University, Beijing 100083, People’s Republic of China

^b^Beijing Advanced Innovation Centre for Biomedical Engineering, Beihang University, Beijing 102402, People’s Republic of China

^c^National Research Center for Rehabilitation Technical Aids, Beijing 100176, People’s Republic of China

^*^Corresponding author: Xianghui Gong, Yubo Fan

Tel.: 86-10-82339428; Fax: 86-10-82339428

Email address: xhgong@buaa.edu.cn, yubofan@buaa.edu.cn

Address: School of Biological Science and Medical Engineering, Beihang University, Xue Yuan Road No. 37, Haidian District, Beijing 100083, People’s Republic of China.

**Material and methods**

**Evaluating cytotoxic effects of long-term incubation under low concentration of resazurin**

MC3T3-E1 cells were seeded into 96-well plates at a density of 2×10^4^ cells/well and cultured for 24 hours. Then cells were incubated in the media contained 0.1 mM resazurin (100μl/well). After 24, 36, 48 hours of incubation, the resazurin-contained media were removed. Cells were washed three times with PBS and cultured in fresh culture media for 48 hours. Then cell viabilities were measured using MTT assay and normalized with that of control cells which were incubated in the fresh culture media. Each condition was tested in quadruplicate.

**Evaluating effects of resazurin depletion on viable cell estimation**

MC3T3-E1 cells were seeded into 96-well plates at a density of 100, 50, 25, 12.5, 6.25, 3.12, and 1.56×10^4^ cells/well using double dilution method and cultured for 24 hours. Then cells were incubated in the fresh media contained 0.1 mM resazurin (100μl/well). The reduction of absorbance at 605 nm was measured by reading the absorbance of the sample on a Varioskan Flash at the time point of 0, 0.25, 0.5, and 1 hour. Each condition was tested in quadruplicate. Wells contained no cells were used as control.


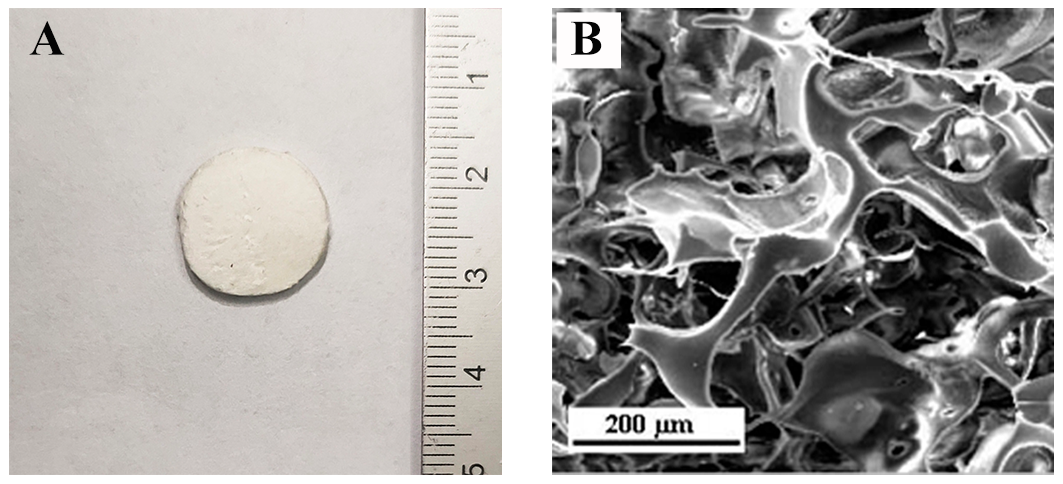


**Fig. S1** The representative image of the PLA scaffold. **A** The representative image of the whole 3D PLA scaffold. **B** The representative SEM photomicrographs of the PLA scaffolds. Scale bar: 200 μm.

**
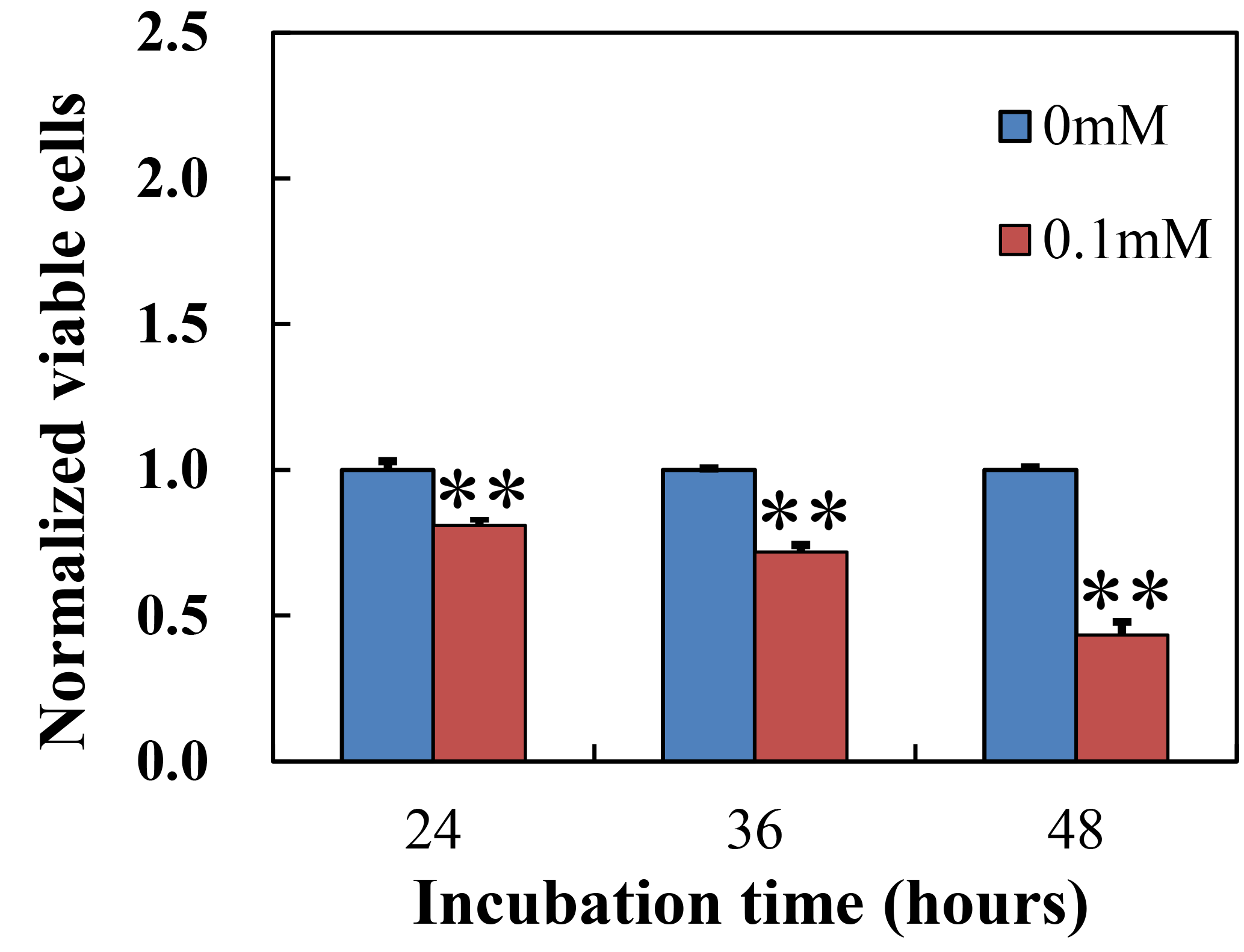
**

**Fig. S2** Cytotoxic effects of long-term incubation with low concentration of resazurin (0.1mM) on MC3T3-E1 cell. ** significant difference between the test group (0.1mM) and the control group (0mM) at p<0.01.


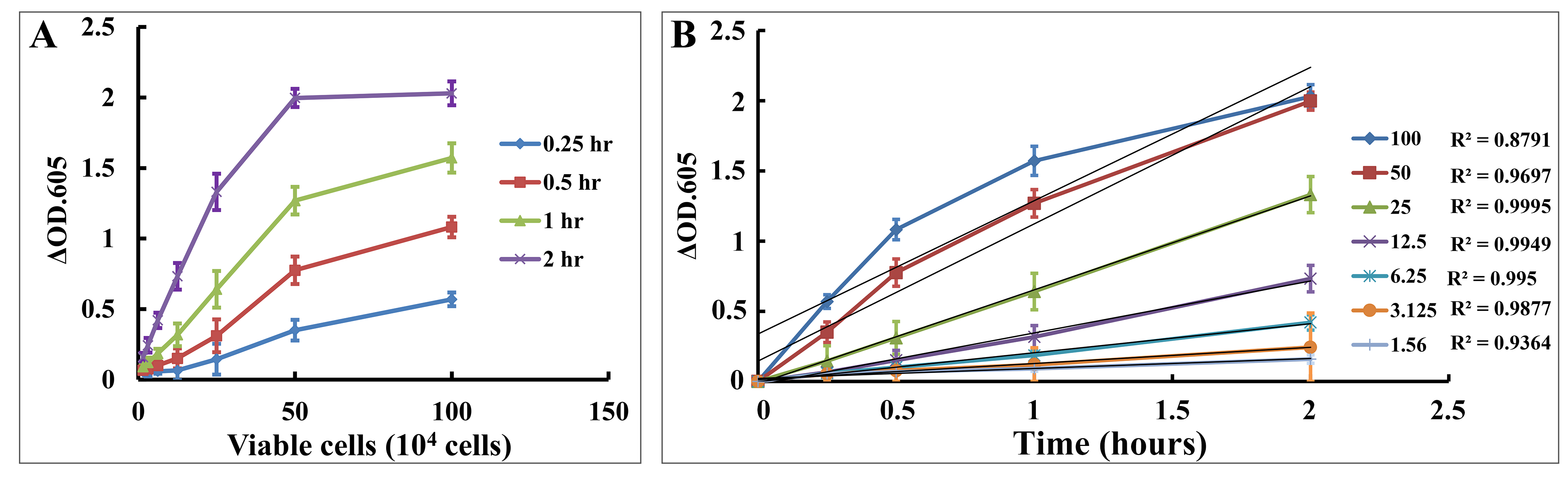


**Fig. S3** Effects of resazurin depletion on viable cells estimation. MC3T3-E1 cells were incubated in 0.1mM resazurin. **A** Absorbance reduction of resazurin as a function of viable cell concentration of MC3T3-E1 and incubation time. **B** The reduction rate of resazurin incubated with different concentrations of MC3T3-E1.
